# In vivo and in vitro metabolism of the designer benzodiazepine, bretazenil: a comparison of pooled human hepatocytes and liver microsomes with postmortem urine and blood samples

**DOI:** 10.1007/s00204-025-04213-x

**Published:** 2025-10-01

**Authors:** Prince S. Gameli, Johannes Kutzler, Laura M. Huppertz, Diletta Berardinelli, Livio Tronconi, Giuseppe Basile, Jeremy Carlier, Francesco P. Busardò, Volker Auwärter

**Affiliations:** 1 Department of Biomedical Sciences and Public Health, Marche Polytechnic University, Via Tronto 10/a, 60126 Ancona, AN Italy; 2 Institute of Forensic Medicine, Forensic Toxicology, Medical Center, University of Freiburg, Faculty of Medicine, Freiburg, Germany; 3 Department of Human Science, European University of Rome, 00163 Rome, Italy; 4 GVM Care & Research, Maria Cecilia Hospital, 480333 Cotignola, Italy

**Keywords:** Metabolite identification, Analytical toxicology, Designer benzodiazepine, Metabolites, High-resolution mass spectrometry

## Abstract

Benzodiazepines are often used with other drugs like opioids, potentially leading to severe intoxications. Bretazenil, an imidazo-tetrahydropyrrolo-1,4-benzodiazepine, developed in the 1980s but never marketed as a medicine, has recently appeared on the illicit drug market. Given its high potency, short elimination half-life, and potential for rapid metabolism, it is essential to identify markers for bretazenil consumption for clinical and forensic purposes. Our study aimed to thoroughly explore bretazenil’s metabolism using web-based in silico prediction tools, in vitro incubation with pooled human liver microsomes and hepatocytes, and to compare these results with authentic postmortem blood and urine samples. The in silico prediction revealed 16 metabolites, mainly formed by hydroxylation (phase I) and further *O*-glucuronidation, sulfation, and methylation (phase II) reactions. High-resolution mass spectrometry and software-aided data processing of in vitro and in vivo samples identified a total of 26 metabolites. Eight metabolites were detected in vitro, 15 in postmortem urine, and 11 in postmortem blood. Hydroxylation on the pyrrolidine ring was predominant. Other phase I reactions, including combinations of dihydroxylation, hydroxylation, reduction, and carboxylation as well as phase II glucuronidation and sulfation on the pyrrolidine ring, imidazole ring, or the *tert*-butyl chain, were also identified. Additionally, we discovered a new benzodiazepine biotransformation pathway via hydroxylation and cysteine conjugation in both human hepatocytes and blood. Due to bretazenil’s extensive metabolism, we recommend hydroxy-bretazenil (B14), reduced hydroxy-bretazenil (B6), and reduced dihydroxy-bretazenil (B1) as significant markers for detecting bretazenil use.

**Supplementary Information:**

The online version contains supplementary material available at 10.1007/s00204-025-04213-x.

## Introduction

**Fig. 1:**
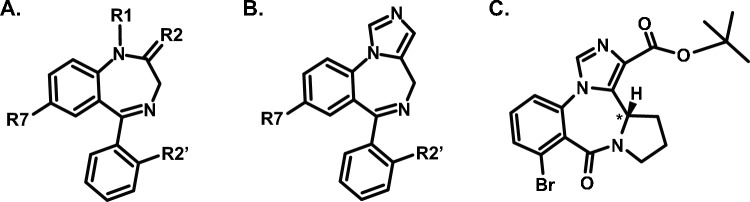
Comparison of generic scheme of typical 1,4-benzodiazepines (**A**), imidazole 1,4-benzodiazepines (**B**) and *S*-bretazenil (**C**)

Bretazenil, also known as “Ro-16-6028” or “bretazenilum”, is a highly potent benzodiazepine that acts as a partial agonist at the gamma aminobutyric acid A (GABA-A) receptor. It was synthesized in the 1980s as a potentially safer anxiolytic, demonstrating lower abuse and addiction potential compared to traditional benzodiazepines such as diazepam and alprazolam (Haigh and Feely 1988; López-Romero et al. 1998). Structurally, bretazenil is an imidazole-type benzodiazepine closely related to imidazenil, midazolam, and the GABA-A receptor antagonist, flumazenil, marketed as Anexeta® and commonly used to reverse benzodiazepine effects (Weerts et al. 2005; Bohnenberger and Liu 2019). Notably, bretazenil possesses a stereocenter at the fused pyrrole-diazepine ring, position 13a (see Fig. 1), resulting in an *S*- and *R*-isomer (Katsifis et al. 1996). The *S*-isomer, patented in 1993 (Amrein et al. 1993), is significantly more potent, exhibiting over 1000-fold greater activity at the GABA-A receptor compared to the *R* counterpart (European Monitoring Centre for Drugs and Drugs Addiction 2021; Environmental Protection Agency 2024).

Preclinical studies suggested that bretazenil has a lower potential for abuse and fewer side effects than other benzodiazepines. Additionally, it is approximately ten times more potent than full agonists such as diazepam (Van Steveninck et al. 1996). However, a randomized, double-blind study by Busto et al. (1994), involving 28 volunteers, found that memory impairment from bretazenil persisted for up to 5 h longer than the effects observed with diazepam and alprazolam. This adverse effect, not previously reported in animal studies, may be attributed to active bretazenil metabolites produced in humans (Busto et al. 1994). This is further supported by bretazenil’s rapid absorption and elimination profile, with half-life ranging from 2 to 4.5 h (Potokar and Nutt 1994; Advisory Council on the Misuse of Drugs 2024). Recently, bretazenil has emerged on the illicit drug market as a new designer benzodiazepine, a subclass of the widely misused new psychoactive substances (NPS). Consequently, it is now monitored by organizations such as the United Nations Office on Drugs and Crime (UNODC) and the European Union Drugs Agency (EUDA, formerly EMCDDA) Early Warning Systems (EWSs) and other national advisory agencies tasked with tracking the emergence and prevalence of NPS (Department of Antidrug Policies 2023; Advisory Council on the Misuse of Drugs 2024; European Union Drugs Agency 2024a; United Nations Office on Drugs and Crime Early Warning Advisory 2024).

Bretazenil was first reported to the EUDA in 2021 after being found among confiscated drug paraphernalia in Sweden and in 2024, Danish authorities seized over 250 bretazenil tablets, along with other NPSs, from a single consignment originating from Germany (European Monitoring Centre for Drugs and Drugs Addiction 2021; European Union Drugs Agency 2024b). Although six bretazenil metabolites were detected in earlier preclinical studies, there is limited data regarding their identification or biotransformational pathways in humans (European Monitoring Centre for Drugs and Drugs Addiction 2021). Given bretazenil’s high potency and its potential involvement in the “benzo-dope” phenomenon—the co-use of benzodiazepines with opioids in illicit drug markets—it is crucial to characterize markers of consumption that can improve the detecting bretazenil-positive cases in clinical and forensic settings.

Analytical toxicology and high-throughput metabolism studies using high-resolution mass spectrometry (HRMS) have proven efficient in identifying markers useful for intoxication cases and informing harm reduction efforts (Carlier et al. 2022; Weston et al. 2022). Therefore, this study explores bretazenil’s metabolism by comparing in silico prediction models, conducting in vitro incubations with pooled human liver microsomes (pHLM) and pooled human hepatocytes, and analyzing postmortem biosamples. By employing a comprehensive approach that combines ultra-high-performance liquid chromatography-high-resolution tandem mass spectrometry (UHPLC-HRMS/MS) with software-aided data processing, we aimed to identify and characterize bretazenil metabolites in humans and consumption markers for bretazenil-positive cases.

## Materials and methods

### Chemicals and reagents

Bretazenil’s pure standard (*S*-epimer) was provided within the EU-ADEBAR plus project (Pulver et al. 2022). Diclofenac was obtained from Sigma Aldrich (Milan, Italy). Stock solutions (1 mg/mL) were prepared in methanol or acetonitrile and stored at − 20 °C. LC–MS grade acetonitrile, water, methanol, and formic acid were purchased from Carlo Erba (Cornaredo, Italy). Reagent-grade ammonium acetate was sourced from Levanchimica (Bari, Italy). Ten-donor-pooled human hepatocytes, thawing medium (TM) and Trypan blue (0.4%) were supplied from Lonza (Basel, Switzerland). Williams’ medium E (WME), HEPES buffer (2-[4-(2-hydroxyethyl)-1-piperazinyl] ethanesulfonic acid), l-glutamine, and β-glucuronidase (*Patella vulgata* L., 100,000–200,000 units/mL) were obtained from Sigma Aldrich. l-Glutamine and HEPES at 2 and 20 mmol/L, respectively, were added to prepare supplemented Williams’ E medium (SWM).

pHLMs (150 donors, 20 mg/mL protein in 250 mM sucrose), NADPH-regenerating solution A (26 mM nicotinamide adenine dinucleotide phosphate (NADP^+^), 66 mmol/L glucose-6-phosphate, and 66 mmol/L magnesium chloride in water), and solution B (40 U/mL glucose-6-phosphate dehydrogenase in 5 mmol/L sodium citrate), with a reductase activity of 0.43 μmol/min/mL, were obtained from Corning (Amsterdam, the Netherlands). Potassium phosphate buffer (0.5 mol/L, pH 7.4), uridine 5′-diphospho-glucuronosyltransferase reaction mixture solution A (25 mmol/L uridine diphosphate-glucuronic acid) and solution B (40 mmol/L magnesium chloride, 250 mmol/L tris(hydroxylmethyl)aminomethane hydrochloride and 0.125 mg/mL alamethicin) were also sourced from Corning.

### In silico metabolites prediction

Bretazenil’s metabolite prediction was performed using the GLORYx web tool. The simplified molecular input line entry system (SMILES) notation for bretazenil was generated using the freeware ACD/ChemSketch (version 2012, build 61438) and entered into the “phase I and II metabolism” option in GLORYx (Stork et al. 2020; De Bruyn Kops et al. 2021). Metabolites with a probability score ≥ 0.20, including both first- and second-generation metabolites (resulting from one and two transformations, respectively), were added to an inclusion list (see Table S1) for subsequent LC-HRMS/MS analysis.

### In vitro incubations and sample preparation

#### Pooled human liver microsomes (pHLM)

The assay was conducted using bretazenil prepared in acetonitrile, following a previously elaborated method (Giorgetti et al. 2023). Incubations for 0.5, 1, and 2 h were performed in triplicates. To quench the reaction, 100 μL of ice-cold acetonitrile and 50 μL of ammonium formate (10 mol/L) were added to each sample. For LC-QToF-MS analysis, 30 μL supernatant was evaporated and reconstituted in 100 μL of mobile phase MPA1 and MPB1 (50:50, v/v). A 5 μL volume was then injected for HPLC-ESI-QToF-MS analysis*.* Blank controls (without bretazenil) and negative controls (without pHLM enzymes) were processed in parallel with the test samples.

#### Pooled human hepatocytes

Bretazenil was incubated with pooled human hepatocytes according to a previous protocol, with minor modifications (Di Trana et al. 2021). Briefly, hepatocytes were thawed in 50 mL of TM, centrifuged for 5 min at 50–100*g*, and the resulting pellets were resuspended in 50 mL TM. After a second centrifugation, the supernatant was discarded, and the pellets resuspended in 2 mL TM. Cell viability was assessed using the Trypan blue exclusion assay, and the volume of SWM was adjusted to 2 × 10^6^ viable cells/mL. A 250 µL aliquot of the hepatocyte suspension was gently mixed with 250 µL of 20 µmol/L bretazenil in SWM in a sterile 24-well plate and incubated for 3 h. A positive control (diclofenac) and negative controls were included to monitor the experiment. The reaction was quenched with 500 μL of ice-cold acetonitrile, followed by centrifugation at 15,000*g* for 10 min. For LC-HRMS/MS analysis, the supernatant was centrifuged, evaporated to dryness, and resulting pellets were reconstituted in mobile phase MPA2 and MPB2 (90:10, v/v). 10 µL of the final supernatant was injected into the LC-HRMS for analysis.

#### Extraction of authentic postmortem samples

For this study, a single bretazenil-positive case detected during routine screening was included to assess the metabolism in vivo. Yohimbine was also identified in the sample during our in-house LC-HRMS/MS screening. No other substance of toxicological relevance was detected. Urine and blood specimens from the postmortem case were stored at − 20 °C prior to analysis and in between analyses. After thawing at room temperature, 100 and 200 µL of acetonitrile were added to 50 and 100 µL of urine and blood, respectively, centrifuged at 15,000*g* for 10 min, the supernatant collected, and dried under nitrogen at 37 °C. The samples were reconstituted with 100 µL MPA2:MPB2 (90:10 v/v) and centrifuged at 15,000*g* for 10 min. A 10 µL volume was injected for LC-HRMS/MS analysis.

To compare conjugated urine metabolites to their unconjugated equivalent, enzymatic hydrolysis with *P. vulgata* was conducted as follows: 50 µL of β-glucuronidase and 5 µL of 10 mmol/L ammonium acetate (pH 5.0) were added to 50 µL of urine and incubated for 90 min at 37 °C. Additionally, as a control of the enzymatic reaction, one incubation was performed with water. The reaction was quenched with 200 µL ice-cold acetonitrile and the mixture subsequently centrifuged for 10 min at 15,000*g*, evaporated, reconstituted with MPA2:MPB2 (90:10 v/v), and 10 µL injected for LC-HRMS/MS studies.

### Instrumental conditions

#### High performance liquid chromatography quadrupole time-of-flight mass spectrometry (HPLC-QToF-MS)

HPLC-QToF-MS was used to identify the pHLM metabolites. The analyses were performed according to a previously published protocol (Zschiesche et al. 2024). Briefly, an Impact II QToF instrument coupled with an Elute HPLC system (Bruker Daltonik, Bremen, Germany) was used. The chromatographic separation was performed on a Kinetex® C18 column (2.6 μm, 100 Å, 100 × 2.1 mm; Phenomenex, Aschaffenburg, Germany), and mobile phases comprised of MPA1 (0.1% formic acid in 10 mmol/L ammonium formate in water) and MPB1 (0.1% formic acid in methanol). Gradient elution at a 0.45 mL/min was as follows: mobile phase MPB1 was held at 20% for the first 1 min, and linearly increased to 25% in 30 s, and then to 50% and 60% in 16.5 min and 3.0 min, respectively. MPB1 finally was increased to 90% within 0.5 min and held for 1.5 min, and initial conditions restored in 0.1 min till the end of the run.

Mass spectrometry data acquisition was conducted using electrospray ionization (ESI) in positive-ionization mode. The spectra were compared to the theoretical metabolites predicted both manually and in silico. Molecular ions matching the expected metabolites were further analyzed using a full-MS and auto-MS/MS scan to record product ion spectra. Each precursor ion underwent a maximum of three MS/MS cycles. The respective ion was then excluded for 0.5 min and reconsidered if the intensity ratio was higher than 2.0 compared to the previous measurement. Metabolites were identified through HPLC-ESI-QToF-MS using manual data processing and had to meet the following criteria for a match: a mass error of the precursor ion of less than 5 ppm, a signal-to-noise ratio greater than 3:1, and a mass tolerance for fragment ions of ± 10 ppm.

#### Ultra-high performance liquid chromatography high resolution mass spectrometry (UHPLC-HRMS)

Hepatocyte incubates and postmortem samples were analyzed using a DIONEX Ultimate 3000 UHPLC system coupled to a Q-Exactive quadrupole orbitrap hybrid high-resolution mass spectrometer (Thermo Scientific, Waltham, Massachusetts, USA), equipped with a heated-electrospray ionization (HESI) source. Chromatographic separation was performed on a Kinetex® Biphenyl column (150 × 2.1 mm, 2.6 μm; Phenomenex, Torrance, California, USA), maintained at 37 °C with a flow rate of 0.4 mL/min. The mobile phases were MPA2 0.1% formic acid in water and MPB2; 0.1% formic acid in acetonitrile. The gradient elution started with 2% mobile phase MPB2 for the initial 2 min and gradually increased to 45%. till 16.5 min. MPB2 was then rapidly raised to 95% within 1 min and held at this level until 20.5 min. Afterwards, the conditions were returned to the initial settings within 0.1 min and maintained for equilibration till the end of the run at 25.0 min.

HESI source parameters were optimized with 1 μg/mL bretazenil solution in mobile phase (MPA2:MPB2, 90:10, v/v). Analyses were conducted in positive- and negative-ionization modes. The source conditions were as follows: spray voltage, ± 3.5 kV; capillary and auxiliary temperature, 300 °C; sheath gas, 5 arbitrary units (AU); auxiliary gas, 50 AU; and S-lens RF level, 50. Data acquisition was from 1 to 18 min using both full-MS scan and data-dependent MS/MS (ddMS^2^) modes. The full-MS scan settings were as follows: automatic gain control (AGC) target, 1 × 10^6^; resolution, 70,000 (full width at half maximum at *m/z* 200); maximum injection time, 256 ms; and scan range, *m/z* 250–700. Settings for ddMS^2^ included: AGC target, 2 × 10^5^; minimum AGC target, 6.5 × 10^2^; resolution, 17,500; maximum injection time, 64 ms; isolation window, *m/z* 1.2; normalized collision energies of 30, 50, and 70 AU with five loops; and dynamic exclusion set at 2.0 s. To ensure accuracy, the mass spectrometer was calibrated prior to analysis using a lock mass list. Additionally, an inclusion list containing the theoretically accurate masses ([M + H]+) of putative metabolites containing either ^79^Br or ^81^Br was used to obtain non-interfered MS/MS spectra, thereby accounting for plausible isotopic distributions.

### Data mining for metabolite identification

HRMS data was processed with Compound Discoverer, v. 3.1.1.12 (Thermo Scientific, Waltham, MA, USA) with modifications based on a previous approach (Di Trana et al. 2021). The workflow (see Table S2) combined both targeted and non-targeted strategies. Ions detected in HRMS/MS were compared to theoretically generated metabolites, using an intensity threshold of 5 × 10^3^ and a mass tolerance of 5 ppm. Subsequently, the HRMS/MS spectra and proposed elemental compositions of both predicted and unpredicted metabolites were compared against the mzCloud database, covering counterfeit drugs, drugs of abuse, and prescription drugs, and ChemSpider, which includes Cayman Chemical database and DrugBank. The intensity thresholds for the database search was set at 10^5^ and mass tolerances of 5 and 10 ppm, respectively, were applied.

## Results

### In silico prediction

From the in silico studies, 16 metabolites were predicted (see Table S3). Specifically, nine first-generation metabolites (pM1–pM9, listed in decreasing order of probability score) and seven second-generation metabolites (pMX-1–pMX-2, in decreasing order of score, where pMX refers to the corresponding first-generation metabolite) were forecasted using a 20% threshold. The most commonly predicted metabolic transformation was hydroxylation at the bromophenyl. These hydroxylated metabolites were further conjugated with glucuronic acid, sulfate, or a methyl group. Other predicted metabolites resulted from hydroxylation, *N*-oxidation, or a combination of *N*-dealkylation and oxidation at the pyrrolidine ring. Additionally, the model predicted *N*-dealkylation that leads to opening of the diazepine ring, and further carboxylation at the bromophenyl ring of bretazenil.

### HRMS fragmentation of bretazenil

To elucidate the structure of bretazenil and its metabolites, bretazenil’s characteristic fragments were generated by ramping collision energies in positive-ionization mode after optimization with 1 µg/mL bretazenil solution, however, the ideal fragmentation conditions for the metabolites may differ. Since bretazenil was undetectable in negative-ionization mode, the same source conditions from positive-ionization mode were applied to negative mode. Unless stated otherwise, the full-MS scan and MS/MS spectra of bretazenil and its metabolites are described in positive-ionization mode.

**Fig. 2:**
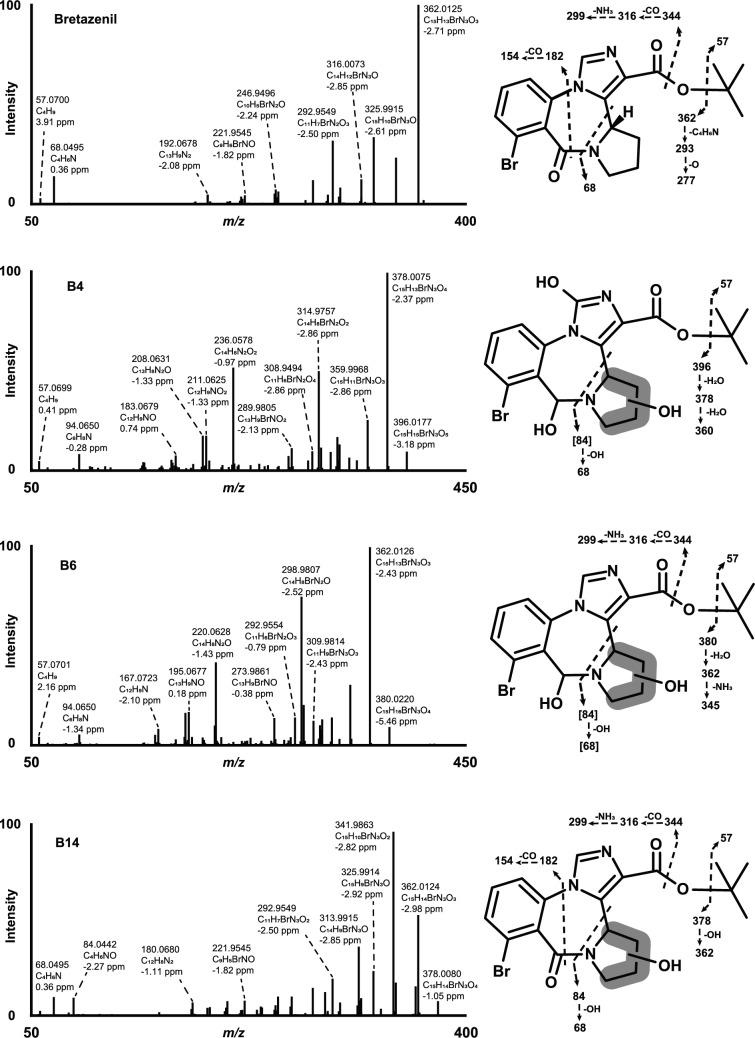
HRMS spectra and proposed fragmentation pattern of bretazenil and its major metabolites; additional fragments and their proposed formation pathway are detailed in the manuscript. Fragment [] were not detected in the product ion spectra. *Gluc* glucuronide

In both in vitro hepatocyte incubations and postmortem samples, bretazenil eluted at 15.78 min, in vitro pHLM at 8.50 min, with a precursor ion at *m/z* 418.0755 (C_19_H_21_BrN_3_O_3_^+^, 1.4 ppm). In the MS/MS spectrum, *m/z* 362.0125 (proposed sum formula; C_15_H_13_BrN_3_O_3_^+^, mass error 2.8 ppm), consistent with the base peak, was produced by cleavage of the *tert*-butyl group (*m/z* 57.0701, 3.9 ppm). Two main fragmentation pathways for *m/z* 362.0125 were identified. In the first pathway, α-cleavage at the carboxylic acid was observed, resulting in the successive loss of a water molecule (− 18.011 u), which could form *m/z* 344.0019 (C_15_H_11_BrN_3_O_2_^+^, 2.9 ppm) and 325.9915 (C_15_H_9_BrN_3_O^+^, 2.6 ppm). Interestingly, the loss of carbon monoxide (− 27.995 u), yielding *m/z* 316.0071 (C_14_H_11_BrN_3_O^+^, 2.9 ppm) directly from *m/z* 344.0019 was also identified as a likely alternate route. Fragment at *m/z* 298.9804 (C_14_H_8_BrN_2_O^+^, 3.5 ppm), neutral loss of ammonia, may have resulted from *m/z* 316.0071 leading to ring contraction., Alternatively, contraction of the 1,4-diazepine ring, a common phenomenon in benzodiazepine fragmentation (Wohlfarth et al. 2017; Gameli et al. 2024b) was observed, leading to a potential loss of the pyrrolidine group (*m/z* 68.0495, 0.4 ppm) from *m/z* 362.0125 and resulting in *m/z* 292.9549 (C_11_H_6_BrN_2_O_3_^+^, 2.4 ppm). Finally, sequential loss of carbon dioxide (− 43.990 u) can form *m/z* 248.9651 (C_10_H_6_BrN_2_O^+^, 2.8 ppm). The fragmentation pattern of bretazenil and its main metabolites identified in this study are presented in Fig. 2.

### Metabolites in pHLMs (HPLC-QToF-MS)

After incubating bretazenil with pHLM for 0.5, 1, and 2 h, a total of eight metabolites were identified. The metabolites, labeled A1 to A8 in order of elution, resulted from hydroxylation (A6, A7, and A8), dihydroxylation (A1, A4, and A5), a combination of hydroxylation and reduction (A2), and carboxylation (A3).

Table 1 provides detailed information on the biotransformation, proposed elemental structures, retention times, exact molecular masses, mass errors (in ∆ppm), and peak areas for bretazenil and metabolites after 0.5, 1, and 2 h of incubation with pHLMs, as determined by HPLC-QToF-MS.

**Table 1:** Biotransformations, elucidated elemental compositions, retention times, accurate molecular masses, and mass errors (∆ppm), and chromatographic peak areas of bretazenil and metabolites after incubation with pooled human liver microsomes (pHLM) for 0.5, 1, and 2 h

ID	RT (min)	Biotransformation	Elemental composition	[M + H]^+^ (*m/z*)	Mass error, ∆ppm	Peak area		
						0.5 h	1 h	2 h
A1	5.65	Hydroxylation (pyrrolidine) + reduction (imidazole)	C_19_H_22_BrN_3_O_4_	436.0864	3.44	2.0 × 10^5^	5.5 × 10^5^	1.2 × 10^6^
A2	5.71	Di-hydroxylation (pyrrolidine + imidazole)	C_19_H_20_BrN_3_O_5_	450.0656	3.99	6.0 × 10^4^	1.4 × 10^5^	2.5 × 10^5^
A3	5.82	Carboxylation (alkyl)	C_19_H_18_BrN_3_O_5_	448.0504	2.24	1.9 × 10^4^	5.9 × 10^4^	9.8 × 10^4^
A4	6.05	Di-hydroxylation (pyrrolidine + imidazole)	C_19_H_20_BrN_3_O_5_	450.0656	3.33	4.8 × 10^5^	1.4 × 10^6^	2.9 × 10^6^
A5	6.55	Di-hydroxylation (pyrrolidine + alkyl)	C_19_H_20_BrN_3_O_5_	450.0656	4.44	8.1 × 10^4^	3.0 × 10^5^	6.9 × 10^5^
A6	7.18	Hydroxylation	C_19_H_20_BrN_3_O_4_	434.0705	4.14	1.7 × 10^6^	3.8 × 10^6^	8.3 × 10^6^
A7	7.25	Hydroxylation	C_19_H_20_BrN_3_O_4_	434.0705	4.37	1.9 × 10^7^	2.0 × 10^7^	2.3 × 10^7^
A8	7.85	Hydroxylation	C_19_H_20_BrN_3_O_4_	434.0704	3.23	7.2 × 10^6^	8.5 × 10^6^	1.0 × 10^7^
Bretazenil	8.50	Parent	C_19_H_20_BrN_3_O_3_	418.0755	3.11	3.4 × 10^7^	3.0 × 10^7^	3.0 × 10^7^

### Metabolites in human hepatocytes and postmortem samples (UHPLC-HRMS-MS)

**Fig. 3:**
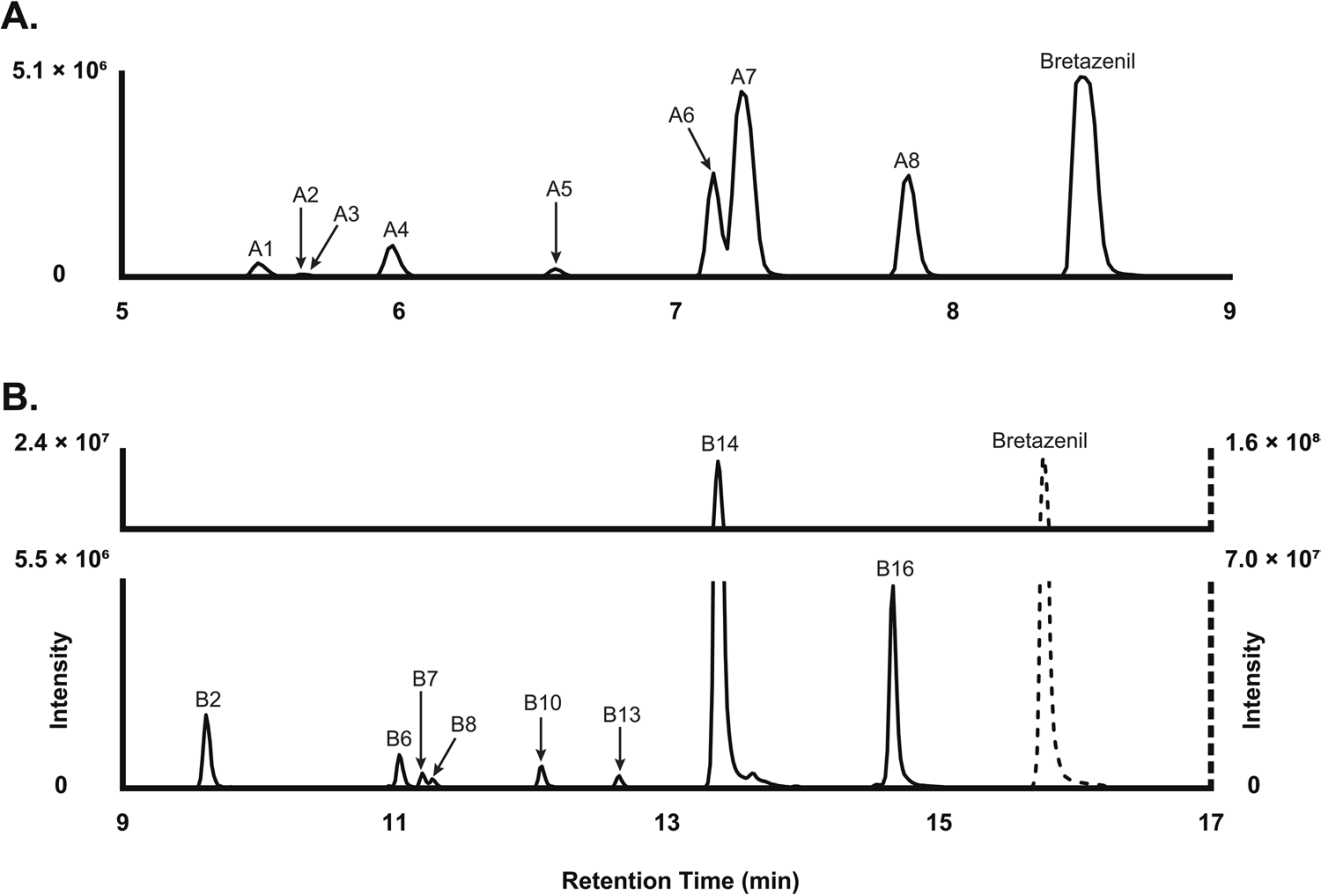
Chromatographic elution of bretazenil and metabolites in in vitro 2 h pooled human liver microsomes (**A**) and 3 h hepatocytes (**B**)

**Fig. 4:**
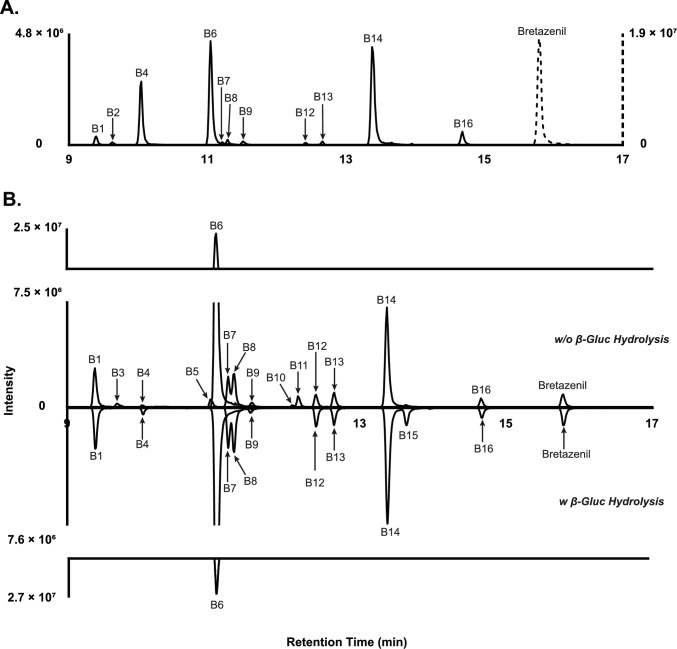
Bretazenil and metabolites identified in postmortem blood (**A**), and hydrolyzed and non-hydrolyzed urine (**B**) detected following UHPLC-HRMS analysis. *β-Gluc* β-glucuronidase

Analysis of hepatocyte incubates and postmortem samples using LC-HRMS revealed 16 metabolites, designated B1–B16 in ascending order of chromatographic retention time. Diclofenac metabolites, 4′-hydroxydiclofenac and diclofenac acyl-glucuronide, were monitored to ensure proper incubation activity with human hepatocytes. Following in vitro incubation with hepatocytes, eight metabolites were detected. The chromatographic elution profiles of bretazenil and its metabolites in 2 and 3 h pHLMs and pooled hepatocytes, respectively, are presented in Fig. 3. In postmortem blood, 11 metabolites were identified. For non-hydrolyzed and hydrolyzed postmortem urine, 14 and 11 metabolites, respectively, were identified. Phase I metabolites were transformed through hydroxylation (B14/A7, B15/A6, and B16/A8), dihydroxylation (B7/A4, B8/A2, and B12/A5), combination of reduction and hydroxylation (B6/A1), reduction and dihydroxylation (B1 and B4), and carboxylation (B13/A3). Phase II metabolites detected included hydroxy-glucuronides (B5, B10, and B11), a dihydroxy-glucuronide (B3), a hydroxy-sulfate (B9), and others (B2). Figure 4 illustrates the chromatograms of bretazenil and its metabolites identified in blood (a) and urine (b), both with and without enzymatic hydrolysis.

A comprehensive overview of the metabolic transformations, elucidated chemical structures, retention times, exact molecular masses, mass errors (in ∆ppm), and peak areas for bretazenil and its metabolites detected in 3 h hepatocytes, postmortem blood, and urine (both non-hydrolyzed and hydrolyzed) using LC-HRMS are presented in Table 2. The MS/MS spectra for all minor metabolites detected in pHLMs, hepatocytes and postmortem samples are provided in Figure S1.

**Table 2:** Biotransformations, elucidated elemental compositions, retention times, accurate molecular masses, and mass errors (∆ppm), and chromatographic peak areas of bretazenil and metabolites following 3 h incubation with human hepatocytes and postmortem blood and urine analysis (hydrolyzed and non-hydrolyzed urine)

ID	RT (min)	Biotransformation	Elemental composition	[M + H]^+^ (*m/z*) [M − H]^−^ (*m/z*)	Mass error (∆ppm)	Peak area, 3 h Hep	Peak area, blood	Peak area, urine	
								Non-H	H
B1	9.38	Di-hydroxylation + reduction	C_19_H_22_BrN_3_O_5_	452.0814 450.0674	− 0.35 0.87	–	1.3 × 10^6^ –	1.0 × 10^7^ 5.4 × 10^5^	9.4 × 10^6^ 6.1 × 10^5^
B2	9.61	Hydroxylation (pyrrolidine) + *O*-cysteine	C_22_H_25_BrN_4_O_5_S	537.0800 535.0656	− 0.34 − 0.05	7.5 × 10^6^ 4.6 × 10^6^	4.3 × 10^5^ 2.9 × 10^5^	–	–
B3	9.68	Di-hydroxylation + *O*-glucuronidation	C_25_H_28_BrN_3_O_11_	626.0974 624.0864	− 0.94 4.74	–	–	1.3 × 10^6^ 8.7 × 10^5^	–
B4	10.02	Di-hydroxylation + reduction	C_19_H_22_BrN_3_O_5_	452.0810 450.0678	− 1.24 1.76	–	9.7 × 10^6^ 1.3 × 10^7^	4.3 × 10^5^ 5.4 × 10^5^	1.4 × 10^6^ 1.3 × 10^6^
B5	10.96	Hydroxylation (pyrrolidine) + *O*-glucuronidation	C_25_H_28_BrN_3_O_10_	610.1016 608.0895	− 2.43 1.60	–	–	4.0 × 10^6^ 1.9 × 10^6^	–
B6	11.04	Hydroxylation (pyrrolidine) + reduction (imidazole)	C_19_H_22_BrN_3_O_4_	436.0861	− 1.25	3.3 × 10^6^	1.6 × 10^7^	8.6 × 10^7^	9.1 × 10^7^
B7	11.20	Di-hydroxylation (pyrrolidine + imidazole)	C_19_H_20_BrN_3_O_5_	450.0652	− 1.58	1.1 × 10^6^	3.7 × 10^5^	6.8 × 10^6^	7.7 × 10^6^
B8	11.29	Di-hydroxylation (pyrrolidine + imidazole)	C_19_H_20_BrN_3_O_5_	450.0648 448.0519	− 2.47 1.21	8.8 × 10^5^ 6.9 × 10^5^	7.2 × 10^5^ –	9.6 × 10^6^ 5.0 × 10^6^	1.1 × 10^7^ 6.1 × 10^6^
B9	11.54	Hydroxylation (alkyl) + *O*-Sulfation	C_19_H_20_BrN_3_O_7_S	514.0277 512.0137	− 0.21 0.87	–	6.7 × 10^5^ 1.0 × 10^5^	1.4 × 10^6^ 2.0 × 10^6^	1.4 × 10^6^ 2.0 × 10^6^
B10	12.07	Hydroxylation (pyrrolidine) + *O*-glucuronidation	C_25_H_28_BrN_3_O_10_	610.1025 608.0897	− 0.97 1.92	2.1 × 10^6^ 1.6 × 10^6^	–	3.8 × 10^5^ 2.2 × 10^5^	–
B11	12.16	Hydroxylation (pyrrolidine) + *O*-glucuronidation	C_25_H_28_BrN_3_O_10_	610.1021 608.0895	− 1.61 1.60	–	–	2.7 × 10^6^ 1.5 × 10^6^	–
B12	12.41	Di-hydroxylation (pyrrolidine + alkyl)	C_19_H_20_BrN_3_O_5_	450.0656	− 0.69	–	3.3 × 10^5^	3.3 × 10^6^	4.3 × 10^6^
B13	12.65	Carboxylation (alkyl)	C_19_H_18_BrN_3_O_5_	448.0497 446.0365	− 1.25 1.78	1.2 × 10^6^ 5.7 × 10^5^	3.9 × 10^5^ –	4.0 × 10^6^ 1.4 × 10^6^	4.1 × 10^6^ 1.5 × 10^6^
B14	13.37	Hydroxylation (pyrrolidine)	C_19_H_20_BrN_3_O_4_	434.0706	− 0.91	8.5 × 10^7^	1.9 × 10^7^	2.8 × 10^7^	2.9 × 10^7^
B15	13.64	Hydroxylation (alkyl)	C_19_H_20_BrN_3_O_4_	434.0727	3.93	–	–	–	5.1 × 10^6^
B16	14.66	Hydroxylation (pyrrolidine)	C_19_H_20_BrN_3_O_4_	434.0699	− 2.52	2.2 × 10^7^	2.1 × 10^6^	2.2 × 10^6^	2.3 × 10^6^
Bretazenil	15.79	Parent	C_19_H_20_BrN_3_O_3_	418.0758	− 0.67	7.0 × 10^8^	8.4 × 10^7^	4.1 × 10^6^	4.8 × 10^6^

HRMS acquisition was in positive- and negative-ionization mode (where applicable)

### Phase I metabolites

#### Hydroxylation

B14, B15, and B16 eluted at 13.37, 13.64, and 12.66 min, respectively, and the protonated molecules were detected at *m/z* 434.0704, corresponding to + 15.995 u mass shift from bretazenil, which is indicative of oxidation. The MS/MS spectrum of B14 closely resembled that of bretazenil. Fragment ion at *m/z* 378.0080 (C_15_H_13_BrN_3_O_4_^+^, 1.1 ppm) suggests a loss of the *tert*-butyl group. Subsequently, the loss of a water molecule can yield *m/z* 359.9967 (C_15_H_11_BrN_3_O_3_^+^, 3.1 ppm), while a second water loss produced the most abundant ion at *m/z* 341.9863 (C_15_H_9_BrN_3_O_2_^+^, 2.9 ppm). Additional ions at *m/z* 344.0019 (C_15_H_11_BrN_3_O_2_^+^, 2.9 ppm), *m/z* 316.0071 (C_14_H_11_BrN_3_O^+^, 2.9 ppm), and *m/z* 292.9549 (C_11_H_6_BrN_2_O_3_^+^, 2.4 ppm) likely represent diazepine ring contraction, consistent with bretazenil’s fragmentation pattern. Notably, hydroxylation likely occurs at the pyrrolidine ring, as indicated by the fragment at *m/z* 84.0442 (C_4_H_6_NO^+^, − 2.3 ppm).

B15 was detected after β-glucuronide hydrolysis in a postmortem urine specimen. The presence of ions at *m/z* 362.0125 (base peak), *m/z* 344.0047, *m/z* 316.0086, and *m/z* 68.0499 suggests that both the core structure and the pyrrolidine ring remain unaltered. Furthermore, fragments at *m/z* 292.9562, *m/z* 276.9619, and *m/z* 248.9663, which correspond to diazepine contraction, were observed at intensities similar to the parent. These findings, combined with the absence of the ion at *m/z* 57.0701, strongly indicate that hydroxylation occurs at the *tert*-butyl group.

In the spectrum of B16, *m/z* 378.0080 (C_15_H_13_BrN_3_O_4_^+^), corresponding to a *tert*-butyl cleavage of a hydroxy-bretazenil metabolite, was not observed. However, two subsequent water losses can yield ions at *m/z* 359.9965 (C_15_H_11_BrN_3_O_3_^+^, 3.6 ppm) and the base peak at *m/z* 341.9863 (C_15_H_9_BrN_3_O_2_^+^, 2.8 ppm). The ion at *m/z* 313.9916 (C_14_H_9_BrN_3_O^+^, 2.2 ppm) may result from a further carbon monoxide loss (− 27.995 u), followed by successive hydrogen cyanide (− 27.011 u) losses, producing ions at *m/z* 286.9807 (C_13_H_8_BrN_2_O^+^, 2.6 ppm) and *m/z* 259.9699 (C_12_H_7_BrNO^+^, 2.3 ppm), indicating diazepine ring contraction. Hydroxylation of B16 thus likely occurs at the pyrrolidine ring, as characterized by the water loss from *m/z* 359.9965.

#### Di-hydroxylation

B7, B8, and B12 had ions at *m/z* 450.0658, eluting at 11.20, 11.29, and 12.41 min, respectively. The observed mass shift, + 32.990 u, indicates that these metabolites are di-hydroxylated.

For B7, the presence of fragment ions at *m/z* 84.0442 (C_4_H_6_NO^+^, 2.3 ppm), *m/z* 359.9967 (C_15_H_11_BrN_3_O_3_^+^, 3.1 ppm), the base peak at *m/z* 341.9862 (C_15_H_9_BrN_3_O_2_^+^, 3.2 ppm), *m/z* 332.0029 (C_14_H_11_BrN_3_O_2_^+^, < 0.1 ppm), and *m/z* 378.0066 (C_15_H_13_BrN_3_O_4_^+^, 4.5 ppm) are consistent with hydroxylation occurring on the pyrrolidine ring. Furthermore, the fragment at *m/z* 378.0066 suggests that a second hydroxylation may take place on the *tert*-butyl, due to the loss of a hydroxylated *tert*-butyl chain.

Similarly, B8 likely undergoes hydroxylation at the pyrrolidine ring, as indicated by the presence of the fragment at *m/z* 84.0442. The fragment at *m/z* 394.0022 (C_15_H_13_BrN_3_O_5_^+^, 2.8 ppm) can result from the loss of a *tert*-butyl chain, suggesting that this chain is not hydroxylated. Subsequent water loss may produce the fragment at *m/z* 375.9917 (C_15_H_11_BrN_3_O_4_^+^, 2.8 ppm). Notably, B8’s base peak at *m/z* 292.9547 (C_15_H_11_BrN_3_O_3_^+^, 2.1 ppm) suggests diazepine ring contraction following the *tert*-butyl chain, and a potentially dihydroxylated pyrrolidine ring.

For B12, the most abundant fragment was detected at *m/z* 341.9863. Additional fragment ions at *m/z* 313.9915, *m/z* 286.9818, *m/z* 259.9714, which are also observed in the MS/MS spectrum of B16, support hydroxylation at the pyrrolidine ring. The presence of fragments at *m/z* 359.9965 (C_15_H_11_BrN_3_O_3_^+^, 3.6 ppm) and *m/z* 341.9863 (C_15_H_9_BrN_3_O_2_^+^, 2.9 ppm) further suggests that the second hydroxylation likely occurs at the *tert*-butyl chain.

#### Hydroxylation(s) and combinations

B6, found at 11.04 min, with an ion at *m/z* 436.0864, was shifted by + 18.011 u relative to bretazenil. This mass shift suggests a combined hydroxylation and reduction reactions. A loss of the unaltered *tert*-butyl chain was observed at *m/z* 380.0244 (C_15_H_15_BrN_3_O_4_^+^, − 1.1 ppm). From this fragment, two consecutive water losses produce *m/z* 362.0124 (C_15_H_13_BrN_3_O_3_^+^, 3.0 ppm), B6’s base peak, and *m/z* 344.0026 (C_15_H_11_BrN_3_O_2_^+^, 0.9 ppm). A further loss of carbon monoxide from *m/z* 344.0026 can yield *m/z* 316.0065 (C_14_H_11_BrN_3_O^+^, 4.7 ppm). Alternatively, a dihydropyrrole and diazepine ring contraction can result in *m/z* 298.9805 (C_14_H_8_BrN_2_O^+^, 3.0 ppm). Additional cleavage of a bromine radical can produce the radical cation at *m/z* 220.0621 (C_14_H_8_N_2_O^+·^, 4.5 ppm), and a subsequent carbon monoxide loss can lead to *m/z* 192.0678 (C_13_H_8_N_2_^+·^, 2.1 ppm). Overall, the fragmentation pathway of B6 indicates that hydroxylation occurs at the pyrrolidine ring, while the reduction most likely takes place at the keto group of the diazepine core substructure.

B4, eluting at 10.02 min, showed an ion at *m/z* 452.0810 (C_19_H_22_BrN_3_O_5_^+^, 1.2 ppm). The mass difference of + 34.0054 u from bretazenil suggests a reduction of the keto group along with two hydroxylations. A loss of the unaltered *tert*-butyl chain can yield *m/z* 396.0177 (C_15_H_15_BrN_3_O_5_^+^, 3.3 ppm), ruling out this alkyl chain as the site of hydroxylation. Two subsequent water losses from *m/z* 396.0177 can generate the base peak at *m/z* 378.0075 (C_15_H_13_BrN_3_O_4_^+^, 2.4 ppm) and a further fragment at *m/z* 359.9968 (C_15_H_11_BrN_3_O_3_^+^, 2.8 ppm), indicating hydroxylation at the pyrrolidine ring. As observed for B6, contraction of the dihydropyrrole and diazepine ring from *m/z* 359.9968 can yield *m/z* 314.9757 (C_14_H_8_BrN_2_O_2_^+^, 2.2 ppm). A further cleavage of a bromine radical can result in the radical cation *m/z* 236.0578 (C_14_H_8_BrN_2_O_2_^+·^, 0.8 ppm), and an additional loss of carbon monoxide can produce *m/z* 208.0631 (C_13_H_8_N_2_O^+·^, < 0.1 ppm). In summary, B4’s fragment pattern indicates hydroxylation at both the pyrrolidine and the imidazole rings. The reduction most likely occurs at the keto group diazepine core, consistent with the observations for B6.

#### Carboxylation

B13, detected at 12.65 min, exhibited an ion at *m/z* 448.05188 (C_19_H_19_BrN_3_O_5_^+^, − 3.5 ppm). The mass difference of + 29.979 u from bretazenil indicates carboxylation. The most intense peak at *m/z* 362.0130, along with fragment ions at *m/z* 344.0033, 325.9910, 316.00790, and 68.0497, are all consistent with unaltered fragments of the bretazenil core structure. This pattern strongly suggests that the biotransformation most likely occurs at the *tert*-butyl chain.

### Phase II metabolites

#### Hydroxylation(s) and *O*-glucuronidation

B5, B10, and B11 were detected at 10.96, 12.07, and 12.16 min, respectively, and exhibited an ion at *m/z* 610.1030, corresponding to + 192.028 u mass shift from bretazenil. This mass shift indicates a hydroxylation (+ O, + 15.995 u) and further glucuronide conjugation (+ C_6_H_8_O_6_, + 176.033 u).

B5 was identified as a conjugate formed after pyrrolidine hydroxylation, similar to metabolite B16. This identification is supported by their comparable MS/MS fragmentation spectra. Specifically, both B5 and B16 displayed fragment ions at *m/z* 359.9979, 341.9864, 313.9918, 180.0687, and 84.0438.

MS/MS spectra of B10 and B11 were similar to that of B14 and B15, respectively. Furthermore, the chromatographic elution suggests the two hydroxy-bretazenil glucuronides, B10 and B11, are phase II glucuronidation products of B14 and B15, respectively. The identification of these metabolites as *O*-glucuronide conjugates is further supported by the presence of fragment ions at *m/z* 159.0285 (C_6_H_7_O_5_^+^, − 1.9 ppm), 131.0342 (C_5_H_7_O_4_^+^, 2.4 ppm), and 85.0282 (C_4_H_5_O_2_^+^, − 2.4 ppm).

Finally, B3 was detected at 9.68 min with an ion at *m/z* 626.0974. The observed mass shift of B3, + 208.0219 u, from bretazenil corresponds to dihydroxylation (+ 2O, + 31.995 u), and glucuronidation (+ C_6_H_8_O_6_, + 176.0326 u). The MS/MS spectrum of B3 with fragment ions at *m/z* 375.9930, 332.0032, 292.9552 (most intense), and 267.0854, closely matches that of B8. Therefore, B3 is identified as a glucuronide conjugate of B8.

### Other biotransformations

B2 eluted at 9.61 min with an ion at *m/z* 537.0800 (C_22_H_25_BrN_4_SO_5_^+^, − 0.3 ppm). B2 and B16 shared several fragment ions in their respective fragmentation spectra. This similarity suggests that B2 may arise from a conjugation reaction of B16. B2 exhibited a mass shift of + 119.0036 u relative to bretazenil, which corresponds to hydroxylation (+ O, + 15.995 u) and possibly the addition of an acyl-cysteine residue (+ C_3_H_5_NOS, + 103.0086 u).

B9 eluted at 11.54 min and produced an ion at *m/z* 514.0277. This compound showed a mass shift of + 95.9522 u from bretazenil, consistent with hydroxylation and sulfation. The most abundant fragment ion of B9 appeared at *m/z* 362.0130. Additionally, the fragmentation spectrum of B9 closely resembled that of B15, as indicated by the presence of main fragments at *m/z* 325.9934, 292.9551, 276.9616, and 68.0494. Therefore, it is likely that B9 arises from the hydroxylation (+ O, 15.995 u) of B15, followed by a subsequent sulfation reaction (+ SO_3_, + 79.9572 u). Notably, B9 exhibited a peak area approximately 30% higher in negative-ionization mode compared to positive-ionization mode.

## Discussion

**Fig. 5:**
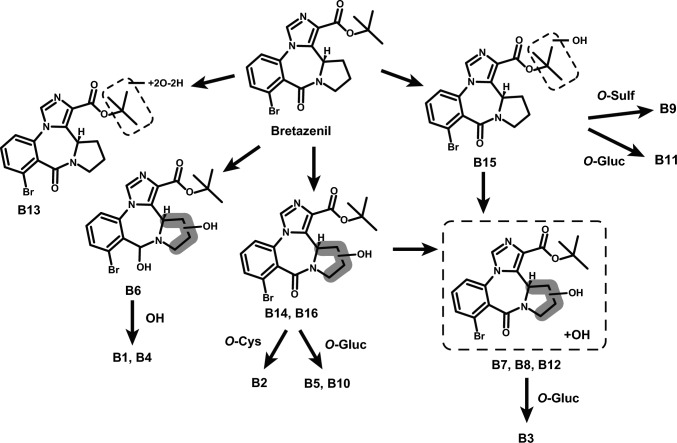
Bretazenil’s proposed biotransformational pathway in human. *Gluc* glucuronide, *Sulf* sulfate, *Cys* cysteine

In the present study, we identified metabolites of bretazenil employing the in silico GLORYx prediction toolkit (16), in vitro pooled HLM (8), pooled human hepatocytes (8), and in vivo authentic biospecimens, blood (11), and urine (15), to holistically map bretazenil’s biotransformations with the aim of proposing consumption markers useful in analytical toxicology. Figure 5 depicts the proposed metabolic fate of bretazenil.

### In silico GLORYx metabolite prediction

To prevent predicting a large amount of unlikely metabolites of bretazenil, a general tendency of in silico prediction tools, a threshold was set at 20%. Nonetheless, all first- and second-generation metabolites predicted in silico in our study had a probability score < 30%. A similar result was reported for flubrotizolam (Gameli et al. 2024b), which presupposes in silico prediction tools may need further fine-tuning, at least for designer benzodiazepines, to improve the accuracy of results. Hydroxylation at the bromophenyl ring was the transformation predicted with the highest probability. However, our in vitro and in vivo studies point to hydroxylation predominantly occurring at the pyrrolidine ring, consistent with pM4 and pM7 predicted in silico. Phase II reactions also tend to follow hydroxylation at the pyrrolidine ring, although sulfation following hydroxylation at the *tert*-butyl chain was detected in blood and urine as opposed to occurring at the bromophenyl ring or the pyrrolidine ring (pM7-1), as predicted in silico. In the current study, in silico metabolite forecasting was employed mainly for optimizing HRMS data acquisition and software-aided data mining.

### In vitro and in vivo metabolism, a comparison

Pooled in vitro human microsomes and hepatocytes incubated with bretazenil resulted in eight metabolites. All eight metabolites were detected in pHLM incubations after 0.5, 1, and 2 h with increasing order of intensity. On the contrary, an *O*-glucuronidated metabolite (B10), and possibly a new biotransformational pathway for benzodiazepines was identified after 3 h hepatocyte incubation with bretazenil, demonstrating that human hepatocytes can comprehensively simulate phase I and II metabolism of xenobiotics. Nonetheless, pHLM is an inexpensive in vitro model and can provide phase I metabolism data in a timely manner that is crucial to monitoring newly emerging drugs and thus should not be overlooked (Zschiesche et al. 2024). A limitation of the study is the use of distinct LC gradients for the HLMs versus the hepatocytes and human specimen analyses, which may complicate direct retention time comparison. Nevertheless, metabolite identification and annotation were achieved with MS/MS fragmentation patterns, ensuring consistency across the different systems. Additionally, the relatively rapid elution kinetics utilized in HLM samples may be optimized in future studies for routine clinical and forensic applications where time efficiency is essential.

In vitro metabolism*,* especially in the human hepatic cell line model, replicated authentic postmortem samples accurately. However, chromatographic peak area differences were observed. Notably, lower levels of B6 and higher peak areas of B14 and B16 were found in hepatocytes. This discrepancy may stem from extrahepatic metabolism, additional pharmacokinetic processes, or stability differences between in vitro hepatocytes and postmortem samples, which should be taken into account when interpretating and comparing data across metabolic models.

Results demonstrated first-generation metabolites were predominantly transformed through hydroxylation occurring mainly at the pyrrolidine ring, followed by other combinations of phase I reactions and subsequent glucuronidation. Such transformations typically occurring at the carbon 3 position of the diazepine ring have been reported for 1,4-benzodiazepines such as phenazepam, diclazepam, and flubromazepam (Moosmann et al. 2013, 2014; Crichton et al. 2015; Gameli et al. 2024a). Interestingly, *N*-glucuronidation detected for triazole-type designer benzodiazepines, including flualprazolam (Wagmann et al. 2020), and even NPS from the 1,4-benzodiazepine class, such as nifoxipam (Pettersson Bergstrand et al. 2019), were not detected in this study. This exemplifies the need for analytical toxicologists to take caution in juxtaposing the metabolism of analogs, especially when proposing markers of consumption. Under the present analytical conditions, however, we were unable to determine the exact biotransformational positions for some metabolites and further experiments will be necessary to verify the tentative sites of these biotransformations.

Interestingly, results following urine hydrolysis showed discrepancies between proposed glucuronide conjugates and observed peak areas. B5 was annotated as a glucuronide of B16 based on their similar MS/MS fragmentation, however, B16 did not significantly increase post-hydrolysis. A similar concept may be applied to B10 and B14, albeit B14’s high chromatographic area may mask any such changes. Conversely, B4 was identified after hydrolysis despite no identified conjugated precursors. Taken together, these results suggest a phase II glucuronide conjugate may not have formed (for B4), alternate pathways, or inefficiency in *β*-glucuronide hydrolysis, warranting further investigation.

### Cysteine conjugation—a new biotransformational pathway?

B2 was identified after incubation with pooled hepatocytes for 3 h and in postmortem blood. Considering the retention time and peak shape of B2, it is definitely not an adduct or the result of in-source fragmentation from the other metabolites detected. The mass shift from the parent, as previously elaborated, is consistent with an acyl-cysteine conjugation following pyrrolidine ring hydroxylation.

Cysteine conjugates predominantly result from the mercapturic acid pathway following glutathionylation and play a key role in the detoxification of xenobiotics (Cooper et al. 2010), although these residues can be cytotoxic when electrophilic substitution is skipped (Hayden and Stevens 1990; Rousar et al. 2023). In the present experiments, however, fragments corresponding to glutathione or cysteine–glycine conjugates were not detected. This is not uncommon, as a similar cysteine conjugation reaction was previously reported and elaborated for xenobiotics and other drugs such as acetaminophen and even doping agents (Fischer et al. 1985; Cooper et al. 2010; Busardò et al. 2022). It is likely that a direct cysteine conjugation following hydroxylation renders bretazenil more hydrophilic and therefore aids its elimination from the body as bretazenil undergoes extensive metabolism. One the other hand, B2 may be a reactive intermediate within the glutathionylation pathway. Mizuno et al. (2009) previously demonstrated that nitro containing 1,4-benzodiazepines, such as flunitrazepam and nimetazepam, can generate toxic intermediates when incubated with HepRG cells, as observed following a glutathione adduct trapping experiment (Mizuno et al. 2009), suggesting B2 may exhibit a similar toxic profile. All in all, this newly detected benzodiazepine biotransformation pathway warrants further in-depth studies to elucidate its formation and potential toxicological implications.

### Markers of consumption for bretazenil

From the present study, B6 (reduced-hydroxy bretazenil) and B14 (hydroxy bretazenil), with relatively similar peak areas, were the most intense metabolites based on chromatographic peak area in postmortem blood samples. However, the peak area of B6 was about 16% less than that of B14. Detecting bretazenil along with B6 and B14 in blood should be indicative of bretazenil consumption.

On the contrary, B6 had more than 60% the intensity of B14 in urine. Bretazenil’s chromatographic peak area was about 10 times less than B6, demonstrating bretazenil may undergo extensive hepatic metabolism. However, this rapid biotransformation likely yields bioactive metabolite(s) that may contribute to the pharmacological profiles of bretazenil as earlier hypothesized (Busto et al. 1994). It is also important for mandated early warning advisories and legislative instrument to be on the lookout for such active metabolites that may re-emerge on the NPS market as is the case for desalkylgidazepam and 3-hydroxyphenazpeam (Moosmann et al. 2013, 2014; Crichton et al. 2015; Gameli et al. 2024a). From our study, we therefore recommend B6, B14, and B1 (reduced di-hydroxy bretazenil), also found with a higher intensity than bretazenil, as consumption markers when analyzing urine samples.

## Conclusion

Designer benzodiazepines are often involved in polydrug intoxication cases and have become a substantial part of the NPS phenomenon. Identifying consumption markers for these psychoactive agents is therefore paramount to clinicians and forensic toxicologists.

We comprehensively mapped the metabolic fate of bretazenil, a partial GABA-A receptor agonist, using pooled human hepatocytes and liver microsomes, and compared the results to the findings from postmortem intoxication cases. In total, 16 metabolites were detected using high-resolution mass spectrometry, and markers of bretazenil consumption were proposed for blood and urine samples. Results from the in vitro assays were comparable to in vivo human metabolism. Furthermore, we potentially identified a new benzodiazepine metabolism pathway, although additional experimentation and instrumental analysis will be necessary to explore this further.

## Supplementary Information

Below is the link to the electronic supplementary material.

Supplementary file1 (EPS 2005 KB)

Supplementary file2 (EPS 1914 KB)

Supplementary file3 (EPS 743 KB)

Supplementary file4 (DOCX 21 KB)

Supplementary file5 (DOCX 15 KB)

Supplementary file6 (DOCX 18 KB)

## Data Availability

Derived data supporting the findings of this study are available from the corresponding author upon request.
