# Supplementary material for: In vivo and in vitro metabolism of the designer benzodiazepine, bretazenil: a comparison of pooled human hepatocytes and liver microsomes with postmortem urine and blood samples
Source: Arch Toxicol. 2025 Oct 1;100(1):259–73. doi: 10.1007/s00204-025-04213-x (PMC12858478; doi:10.1007/s00204-025-04213-x)
Supplement: Supplementary file 4 — Supplementary file4 (DOCX 21 KB) [file 204_2025_4213_MOESM4_ESM.docx]

**Table S1.** Bretazenil Inclusion List for HRMS/MS data acquisition

| **Transformation** | **Molecular Formula** | **[M^79^Br + H]^+^ *m/z*** | **[M^81^Br + H]^+^ *m/z*** | **[M^79^Br - H]^-^ *m/z*** | **[M^81^Br - H]^-^ *m/z*** | **Comments** |
| --- | --- | --- | --- | --- | --- | --- |
| Bretazenil | C_19_H_20_BrN_3_O_3_ | 418.0760 | 420.0740 | 416.0615 | 418.0595 | Parent |
| +O | C_19_H_20_BrN_3_O_4_ | 434.0710 | 436.0690 | 432.0564 | 434.0544 | Oxidation [Hydroxylation] or Diazepine opening to aldehyde |
| -4C -8H | C_15_H_12_BrN_3_O_3_ | 362.0135 | 364.0114 | 359.9989 | 361.9969 | *O*-Dealkylation to Carboxyl |
| -2H +2O | C_19_H_18_BrN_3_O_5_ | 448.0503 | 450.0482 | 446.0357 | 448.0337 | Carboxylation (Aliphatic) |
| +2O | C_19_H_20_BrN_3_O_5_ | 450.0659 | 452.0639 | 448.0514 | 450.0493 | Oxidation [Di-hydroxylation] |
| +6C +8H +7O | C_25_H_28_BrN_3_O_10_ | 610.1031 | 612.1010 | 608.0885 | 610.0865 | Oxidation + Glucuronidation |
| +2C +6O | C_21_H_20_BrN_3_O_9_ | 538.0456 | 540.0435 | 536.0310 | 538.0290 | *O*-Dealkylation to Carboxyl + Glucuronidation |
| +6C +9H +6O | C_25_H_29_BrN_3_O_9_^+^ | 594.1082 | 596.1061 | - | - | *N*-Glucuronidation [Charged] |
| -4C -8H +O | C_15_H_12_BrN_3_O_4_ | 378.0084 | 380.0064 | 375.9938 | 377.9918 | *O*-Dealkylation to Carboxyl + Oxidation [Hydroxylation] |
| -2H +O | C_19_H_18_BrN_3_O_4_ | 432.0553 | 434.0533 | 430.0408 | 432.0388 | Oxidation [Ketone] |
| +H -Br | C_19_H_21_N_3_O_3_ | 340.1656 | - | 338.1510 | - | Debromination |
| +2H | C_19_H_22_BrN_3_O_3_ | 420.0917 | 422.0897 | 418.0772 | 420.0751 | Reduction |
| -2H | C_19_H_18_BrN_3_O_3_ | 416.0604 | 418.0584 | 414.0459 | 416.0438 | Desaturation |
| +2H +2O | C_19_H_22_BrN_3_O_5_ | 452.0816 | 454.0795 | 450.0670 | 452.0650 | Dihydrodiol formation |
| +2C +3H +O | C_21_H_23_BrN_3_O_4_^+^ | 460.0867 | 462.0846 | - | - | *N*-Acetylation [Charged] |
| +4O +S | C_19_H_20_BrN_3_O_7_S | 514.0278 | 516.0258 | 512.0132 | 514.0112 | Oxidation + *O*-Sulfation |
| -4C -8H +3O +S | C_15_H_12_BrN_3_O_6_S | 441.9703 | 443.9683 | 439.9557 | 441.9537 | *O*-Dealkylation to Carboxyl + *O*-Sulfation |
| +6C +8H +8O | C_25_H_28_BrN_3_O_11_ | 626.0980 | 628.0960 | 624.0834 | 626.0814 | Oxidation [Di-hydroxylation] + *O*-Glucuronidation |
| +2C +7O | C_21_H_20_BrN_3_O_10_ | 554.0405 | 556.0384 | 552.0259 | 554.0239 | *O*-Dealkylation to Carboxyl + Oxidation + *O*-Glucuronidation |
| +H +O -Br | C_19_H_21_N_3_O_3_ | 356.1605 | - | 354.1459 | - | Oxidation [Hydroxylation] + Debromination |
| -4C -7H -Br | C_15_H_13_N_3_O_3_ | 284.1030 | - | 282.0884 | - | *O*-Dealkylation to Carboxylation + Debromination |
| -C -O +2H | C_18_H_22_BrN_3_O_2_ | 392.0968 | 394.0948 | 390.0823 | 392.0802 | Dealkylation |
| -5C -8H -2O | C_14_H_12_BrN_3_O | 318.0236 | 320.0216 | 316.0091 | 318.0071 | Alcohol dehydrogenation [Loss of ethylpriopionate chain] |
| -C +2H | C_18_H_22_BrN_3_O_3_ | 408.0917 | 410.0897 | 406.0772 | 408.0751 | Oxidation + Dealkylation |
| +C10 +14H +3N +6O +S -Br | C_29_H_34_N_6_O_9_S | 643.2181 | - | 641.2035 | - | Glutathione Conjugation |
