# Supplementary material for: In vivo and in vitro metabolism of the designer benzodiazepine, bretazenil: a comparison of pooled human hepatocytes and liver microsomes with postmortem urine and blood samples
Source: Arch Toxicol. 2025 Oct 1;100(1):259–73. doi: 10.1007/s00204-025-04213-x (PMC12858478; doi:10.1007/s00204-025-04213-x)
Supplement: Supplementary file 5 — Supplementary file5 (DOCX 15 KB) [file 204_2025_4213_MOESM5_ESM.docx]

**Table S2.** Transformations for generating potential phase I and phase II metabolites of Bretazenil in Compound Discoverer data mining software

| Phase I | Desaturation (H2 → ) Dihydrodiol formation ( → H2 O2)  Ketone formation (O → H2) Oxidation ( → O) Oxidative Deamination to Alcohol (H2N → HO) Oxidative Debromination (Br → HO) Reduction ( → H2) Reduction Debromination (Br → H) |
| --- | --- |
| Phase II | Acetylation (H → C2 H3 O) Cysteine conjugation on Br (Br → C3 H6 N O2 S) Cysteine-Glycine Conjugation on Br (Br → C5 H9 N2 O3 S) Glucuronide Conjugation (H → C6 H9 O6) GSH Conjugation on Br (Br → C10 H16 N3 O6 S)  Methylation (H → C H3) Sulfation ( H → H O3 S) |
| Others | Alcohol dehydrogenation (C5 H8 O2 → )  *O*-Dealkylation (C4 H8 → )  *O*-Ring opening (C H2 O → ) |
| Max # Dealkylation | 2 |
| Max # Phase II | 2 |
| Max # of Reactions | 5 |
