# Supplementary material for: In vivo and in vitro metabolism of the designer benzodiazepine, bretazenil: a comparison of pooled human hepatocytes and liver microsomes with postmortem urine and blood samples
Source: Arch Toxicol. 2025 Oct 1;100(1):259–73. doi: 10.1007/s00204-025-04213-x (PMC12858478; doi:10.1007/s00204-025-04213-x)
Supplement: Supplementary file 6 — Supplementary file6 (DOCX 18 KB) [file 204_2025_4213_MOESM6_ESM.docx]

**Table S3.** Predicted bretazenil metabolites (first- and second-generation), transformations, structure, prediction score and SMILES forecasted using web-based GLORYx.

| ***ID*** | ***Transformation*** | ***Structure*** | ***Score (%)*** | ***SMILES*** |
| --- | --- | --- | --- | --- |
| Parent | Bretazenil | C_19_H_20_BrN_3_O_3_ | - | CC(C)(C)OC(=O)c1ncn2c1C1CCCN1C(=O)c1c2cccc1Br |
| pM1 | Hydroxylation | C_19_H_20_BrN_3_O_4_ | 27 | CC(C)(C)OC(=O)c1ncn2c3c(C(=O)N4CCCC4c21)c(Br)ccc3O |
| pM1-1 | + *O*-Sulfation | C_19_H_20_BrN_3_O_7_S | 24 | CC(C)(C)OC(=O)c1ncn2c3c(C(=O)N4CCCC4c21)c(Br)ccc3OS(=O)(O)=O |
| pM1-2 | + *O*-Glucuronidation | C_25_H_28_BrN_3_O_10_ | 23 | OC1OC(Oc2ccc(Br)c3C(=O)N4CCCC4c4n(cnc4C(=O)OC(C)(C)C)c32)C(O)C(C(O)=O)C1O |
| pM2 | Hydroxylation | C_19_H_20_BrN_3_O_4_ | 27 | CC(C)(C)OC(=O)c1ncn2c1C1CCCN1C(=O)c1c(Br)c(O)ccc21 |
| pM2-1 | + *O*-Glucuronidation | C_25_H_28_BrN_3_O_10_ | 26 | O=C(OC(C)(C)C)c1ncn2c1C1CCCN1C(=O)c1c(Br)c(ccc21)OC1OC(O)C(C(O)=O)C(O)C1O |
| pM2-2 | + *O*-Methylation | C_19_H_20_BrN_3_O_4_ | 23 | COc1ccc2n3cnc(C(=O)OC(C)(C)C)c3C3CCCN3C(=O)c2c1Br |
| pM3 | Hydroxylation | C_19_H_20_BrN_3_O_4_ | 27 | CC(C)(C)OC(=O)c1ncn2c1C1CCCN1C(=O)c1c2cc(O)cc1Br |
| pM3-1 | + *O*-Glucuronidation | C_25_H_28_BrN_3_O_10_ | 26 | O=C(OC(C)(C)C)c1ncn2c1C1CCCN1C(=O)c1c2cc(cc1Br)OC1OC(O)C(C(O)=O)C(O)C1O |
| pM3-2 | + *O*-Sulfation | C_19_H_20_BrN_3_O_7_S | 25 | CC(C)(C)OC(=O)c1ncn2c1C1CCCN1C(=O)c1c2cc(cc1Br)OS(=O)(O)=O |
| pM4 | Hydroxylation | C_19_H_20_BrN_3_O_4_ | 25 | CC(C)(C)OC(=O)c1ncn2c1C1CCC(O)N1C(=O)c1c2cccc1Br |
| pM5 | Oxidation | C_19_H_20_BrN_3_O_4_ | 25 | CC(C)(C)OC(=O)c1ncn2c1C(CCC=O)NC(=O)c1c2cccc1Br |
| pM6 | *N*-Oxidation | C_19_H_21_BrN_3_O_4_ | 25 | CC(C)(C)OC(=O)c1ncn2c1C1CCC[N+]1(O)C(=O)c1c2cccc1Br |
| pM7 | Hydroxylation | C_19_H_20_BrN_3_O_4_ | 25 | CC(C)(C)OC(=O)c1ncn2c1C1CC(O)CN1C(=O)c1c2cccc1Br |
| pM7-1 | + *O*-Sulfation | C_19_H_20_BrN_3_O_7_S | 25 | CC(C)(C)OC(=O)c1ncn2c1C1CC(CN1C(=O)c1c2cccc1Br)OS(=O)(O)=O |
| pM8 | Oxidation | C_19_H_18_BrN_3_O_4_ | 25 | CC(C)(C)OC(=O)c1ncn2c1C1CC(=O)CN1C(=O)c1c2cccc1Br |
| pM9 | Diazepine opening & Carboxylation | C_19_H_22_BrN_3_O_4_ | 25 | CC(C)(C)OC(=O)c1ncn(c1C1CCCN1)c1cc(Br)cc(c1)C(O)=O |
